# Comprehensive profiling of gut microbiota in postmenopausal osteoporosis

**DOI:** 10.55730/1300-0144.6187

**Published:** 2026-01-30

**Authors:** Gülsemin ERTÜRK ÇELİK, Sedat YILDIZ, Münevver AKSOY BEKTAŞ, Yaprak ENGİN ÜSTÜN

**Affiliations:** 1 Physical Medicine and Rehabilitation Specialist, Republic of Turkey Ministry of Youth and Sports, Ankara, Turkiye; 2 Physical Therapy and Rehabilitation Private Clinic, Ankara, Turkiye; 3 Department of Obstetrics and Gynecology, University of Health Sciences, Etlik Zübeyde Hanım Women’s Health Training and Research Hospital, Ankara, Turkiye

**Keywords:** Osteoporosis, gut microbiota, postmenopausal women, 16S rRNA sequencing, bone mineral density

## Abstract

**Background/aim:**

Osteoporosis is a multifactorial skeletal disease that predominantly affects postmenopausal women and is characterized by reduced bone mass and an increased risk of fractures. Growing evidence suggests that the gut microbiota plays a pivotal role in bone metabolism through immunological, endocrine, and metabolic pathways, forming the basis of the so-called gut–bone axis. This study aimed to characterize gut microbiota composition in postmenopausal women with osteoporosis compared with healthy controls.

**Materials and methods:**

Forty postmenopausal women aged 55–65 years were classified into osteoporosis (n = 20) and healthy control (n = 20) groups based on dual-energy X-ray absorptiometry T-scores. Fecal samples were collected, and gut microbiota composition was evaluated with full-length 16S rRNA gene sequencing using Oxford Nanopore Technologies.

**Results:**

No significant differences were observed in clinical or demographic characteristics between groups, except for the expected lower bone mineral density (BMD) in the osteoporosis group. Alpha-diversity indices showed no statistically significant differences, although a trend toward reduced microbial richness was observed in the osteoporotic participants. Beta-diversity analysis revealed a modest but significant phylogenetic distinction via weighted UniFrac analysis (p < 0.05). Short-chain fatty acid-producing species, including *Faecalibacterium prausnitzii*, *Anaerostipes faecalis*, and *Lactonifactor longoviformis*, were significantly reduced in the osteoporosis group. *F. prausnitzii* abundance was positively correlated with the femoral neck T-score (r = 0.41, p = 0.018), whereas *Oxobacter pfennigii* showed a negative correlation with lumbar spine BMD (r = −0.43, p = 0.015).

**Conclusion:**

Postmenopausal osteoporosis is associated with a distinct gut microbiota profile marked by reduced antiinflammatory and estrogen-modulating taxa alongside increased proinflammatory species. These alterations may contribute to impaired bone metabolism through disrupted calcium absorption, systemic inflammation, and hormonal dysregulation. These findings further support the gut–bone axis and highlight the potential of gut microbiota as a biomarker and therapeutic target in osteoporosis.

## 1. Introduction

Osteoporosis is a progressive, multifactorial skeletal disorder characterized by decreased bone mass, impaired bone strength, and an increased susceptibility to fractures [1]. It predominantly affects postmenopausal women, in whom estrogen deficiency accelerates bone turnover and disrupts the balance between osteoblast-mediated bone formation and osteoclast-driven resorption [2]. Postmenopausal osteoporosis is a systemic bone metabolic disorder driven by estrogen deficiency, leading to bone loss, microstructural deterioration, and heightened fracture risk [1–3].

Beyond classical risk factors such as age, genetics, nutrition, physical inactivity, hormonal status, and medications, which partially explain osteoporosis development, recent preclinical and clinical research suggests that the gut microbiota may also play a critical role in bone health [4–6]. The gut microbiota exerts systemic effects via immune modulation, maintenance of gut barrier integrity, nutrient absorption, and production of bioactive metabolites, such as short-chain fatty acids (SCFAs), bile acids, and indoles [7,8]. The gut–bone axis describes the bidirectional interactions between the gut microbiota and bone metabolism [9,10]. The gut microbiota is central to the gut–bone axis, influencing bone metabolism via complex hormonal, immune, metabolic, and barrier pathways [9,11].

SCFAs, particularly butyrate, propionate, and acetate, enhance calcium and magnesium absorption, promote regulatory T-cell proliferation, and suppress osteoclastogenesis via epigenetic modulation. They also modulate immune responses, enhance calcium absorption, strengthen gut barrier function, and regulate inflammatory pathway for bone health [10,12,13]. Alterations in gut microbiota are linked to systemic inflammation, impaired nutrient absorption, and reduced SCFA production, all of which may compromise bone integrity [14,15].

Emerging evidence indicates that gut microbiota plays distinct roles in various forms of osteoporosis [16–18]. In postmenopausal osteoporosis, dysbiosis is associated with estrogen metabolism and immune modulation, particularly involving an imbalance in T helper 17 (Th17)/regulatory T (Treg) cells that exacerbates bone loss [19,20]. The concept of the brain–gut–bone axis further highlights how the gut microbiota influences bone remodeling by affecting pathways such as the receptor activator of nuclear factor kappa β ligand/osteoprotegrin, micro-RNAs, and insulin-like growth factor-1 (IGF-1) signaling [17,21]. Gut microbiota alterations can enhance gut permeability, allowing bacterial products like lipopolysaccharides to trigger systemic inflammation, which promotes osteoclast activation and bone resorption. Additionally, common osteoporosis treatments (e.g., bisphosphonates) may interact with the gut microbiota, potentially influencing their efficacy. Nonsteroidal anti-inflammatory drugs, widely used to treat pain in osteoporosis, can disrupt gut microbiota, reduce butyrate production, and impair bone healing [16]. Probiotics, prebiotics, and dietary fiber support gut health and have shown promise in enhancing bone density, reducing bone resorption, and improving bone healing in preclinical models [17].

Despite these promising findings, few human studies have characterized gut microbial shifts associated with osteoporosis using advanced sequencing technologies [16–18]. The gut microbiota could serve as both a biomarker and therapeutic target for osteoporosis and bone healing strategies. It was hypothesized herein that osteoporosis is associated with distinct microbial signatures, including the depletion of beneficial taxa, which may contribute to disease pathophysiology. Therefore, this study aimed to characterize the gut microbiota composition of osteoporotic and healthy postmenopausal women using full-length 16S rRNA sequencing. The secondary objective was to evaluate potential associations between specific microbial taxa and bone mineral density (BMD) parameters.

## 2. Materials and methods

### 2.1. Study design and ethical approval

This observational, cross-sectional study was conducted between January and June 2023 at a tertiary academic center. The study was approved by the institutional ethics review board (TC Ministry of Health Etlik Zübeyde Hanim Gynecology and Pediatrics Training and Research Hospital) and complied with the principles outlined in the Declaration of Helsinki. All participants provided written informed consent prior to enrollment.

### 2.2. Participant selection and grouping

A total of 40 postmenopausal women, between 55 and 65 years of age, were recruited and categorized into two groups: 20 with osteoporosis (T-score ≤ −2.5 standard deviations (SD)) and 20 healthy controls (T-score ≥ −1.0 SD) as determined using dual-energy X-ray absorptiometry (DXA). The World Health Organization stated that a T-score of ≤ −2.5 SD indicates the presence of osteoporosis [22]. A T-score in the range of −1 to −2.5 SD indicates osteopenia, or poor bone mass. Inclusion criteria were being postmenopausal for more than 12 months and the absence of chronic inflammatory or infectious diseases. Exclusion criteria were the use of antibiotics, prebiotics, probiotics, or corticosteroids in the past 3 months; diagnosis of metabolic bone disease other than osteoporosis; having gastrointestinal disorders; malignancy; liver or kidney failure; and current antiosteoporotic treatment.

### 2.3. Clinical and demographic data collection

Detailed medical histories, medication usage, dietary habits, physical activity levels, and menopausal duration were recorded via structured interviews. Anthropometric measurements included height, weight, and body mass index (BMI). BMD was measured at the lumbar spine (L1–L4) and proximal femur using DXA (Hologic Discovery-A; Hologic Inc., Bedford, MA, USA) following International Society for Clinical Densitometry guidelines. T-scores and Z-scores were also recorded.

### 2.4. Fecal sample collection and DNA extraction

Participants were instructed to collect a fresh stool sample using a standardized collection kit provided by the research team. Samples were promptly stored at 4 °C and transported to the laboratory within 4 h, and then aliquoted and preserved at −80 °C until processing. Genomic DNA was extracted from approximately 200 mg of stool using the ZymoBIOMICS DNA miniprep kit (Zymo Research Corp., Irvine, CA, USA) according to the manufacturer’s instructions. DNA quantity and quality were assessed using a NanoDrop ND-1000 spectrophotometer (Thermo Fisher Scientific Inc., Waltham, MA, USA) and Qubit fluorometer (Invitrogen Life Technologies, Waltham, MA, USA).

### 2.5. 16S rRNA gene amplification and Oxford nanopore sequencing

The full-length 16S rRNA gene (regions V1–V9) was amplified using universal primers provided in the SQK-16S024 library preparation kit (Oxford Nanopore Technologies, Oxford, UK). Polymerase chain reactions were performed in triplicate for each sample using high-fidelity Taq polymerase. Sequencing libraries were prepared following the Oxford Nanopore Technologies instructions and loaded onto FLO-MIN106D R9.4.1 flow cells mounted on a MinION Mk1B device. Raw sequencing data were collected using MinKNOW software and converted from FAST5 to FASTQ format using Guppy basecaller. Sequence reads were quality filtered with a Phred score threshold of ≥Q7 and length range 1300–1600 bp. Barcoded samples were demultiplexed, and chimeric sequences were removed prior to downstream analysis.

### 2.6. Bioinformatics and taxonomic classification

Filtered reads were aligned to reference databases (National Center for Biotechnology Information 16S Microbial and Human Microbiome Project) using minimap2 with custom scripts. Amplicon sequence variants (ASVs) were clustered at full-length species-level alignment threshold using VSEARCH. Taxonomic assignments were made from phylum to species level. An ASV abundance matrix was generated, normalized by total sum scaling, and used for diversity analysis. Environmental and low-abundance taxa were filtered using abundance and prevalence thresholds before differential analysis.

### 2.7. Microbial diversity and statistical analysis

A total of 1,391,241 high-quality reads were generated across all the samples with an average of 34,781 reads per sample (range: 21,045–59,842). The average read length was 1450 bp with a median Phred score above Q10. After quality filtering and chimera removal, a total of 2087 unique ASVs were identified across both groups. Rarefaction curves indicated sufficient sequencing depth to capture most of the bacterial diversity. Bacterial diversity analyses were performed in two stages. Alpha diversity indices, including observed ASVs, the Shannon index, Chao1 richness, and Inverse Simpson index, were calculated using the vegan and phyloseq packages in R (v4.3.1) [23,24]. Beta diversity was assessed using Bray–Curtis dissimilarity, Jaccard index, and weighted and unweighted UniFrac distances, and visualized using principal coordinate analysis (PCoA). Intergroup comparisons were performed using parametric (Student’s t) or nonparametric (Mann–Whitney U) tests based on Shapiro–Wilk normality assessments. Categorical variables were compared using chi-squared or Fisher’s exact tests. Differential abundance testing was conducted using the DESeq2 and linear discriminant analysis effect size algorithms. p-values were adjusted using Benjamini–Hochberg false discovery rate correction. p < 0.05 was considered statistically significant.

## 3. Results

### 3.1. Clinical and demographic characteristics

The study included 40 postmenopausal women, divided equally into osteoporotic (n = 20) and healthy control (n = 20) groups. No significant differences were observed between the groups in age, duration of menopause, age at menopause, weight, BMI, height shortening, sleep duration, or defecation habits (p > 0.05 for all) (as shown in the Table). As expected, the BMD, T-scores, and Z-scores at both the femur and spine were significantly lower in the osteoporosis group compared to the control group (p < 0.001 for all), confirming diagnostic classification.

### 3.2. Alpha diversity analysis

Alpha diversity indices, including Shannon diversity, the Simpson index, and observed ASVs, were used to assess within-sample diversity. The mean Shannon index was slightly lower in the osteoporotic group (4.12 ± 0.35) compared to the control group (4.36 ± 0.40), but the difference did not reach statistical significance (p = 0.084). Similarly, the Chao1 richness index showed a trend toward reduced richness in the osteoporotic group but it was not statistically significant (mean: 368 ± 27 vs. 391 ± 30; p = 0.067). No statistically significant difference was observed between the groups in the alpha diversity obtained at any of the taxonomic levels (p > 0.05).

### 3.3. Beta diversity and community composition

Beta diversity analysis was performed using PCoA based on Bray–Curtis, Jaccard, weighted UniFrac, and unweighted UniFrac distances, each capturing different aspects of community dissimilarity. While the Bray–Curtis and Jaccard metrics did not reveal significant separation between the osteoporosis and control groups, the weighted UniFrac distances demonstrated a modest yet statistically significant phylogenetic difference (p = 0.041), suggesting compositional shifts involving evolutionary line-ages rather than gross changes in community structure. This distinction was visualized by the partial clustering observed in the weighted UniFrac PCoA plot.

### 3.4. Taxonomic composition

**Figure 1:**
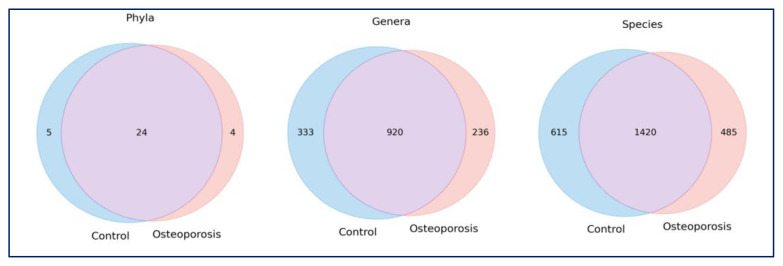
Venn diagrams comparing the gut microbiota composition between healthy controls (blue) and osteoporotic patients (red) at the (a) phylum, (b) genus, and (c) species levels. The overlapping areas represent taxa shared between groups, while those not overlapping indicate taxa unique to each group. A greater proportion of unique taxa was observed at the genus and species levels, indicating more pronounced microbial divergence at finer taxonomic resolutions in osteoporosis.

The ASV numbers obtained in the study were 33 at the phylum level, 1489 at the genus level, and 2520 at the species level. Venn diagram analysis revealed that, at the phylum level, 72.7% of phyla were shared between the groups, with 15.2% unique to the control group and 12.1% unique to the osteoporotic group (Figure 1). At finer taxonomic resolutions, divergence increased; 22.4% of genera and 24.4% of species were unique to the control group, compared to 15.8% of genera and 19.2% of species unique to the osteoporosis group. These findings suggest that while a core microbiome is retained, osteoporosis is associated with significant microbial shifts, particularly at the species and genus levels (Figure 1).

At the phylum level, the dominant bacterial phyla across all the samples were *Bacillota* (formerly *Firmicutes*), comprising 42.7% in the osteoporotic group and 49.3% in the control group. *Bacteroidota* accounted for 36.2% in the control group and 32.1% in the osteoporosis group. Relative abundances of *Pseudomonadota* (10.3% vs. 7.6%) and *Verrucomicrobiota* (3.4% vs. 1.2%) were higher in the osteoporotic group, although not statistically significant. Two phyla—*Acidobacteriota* and *Lentisphaerota*—also demonstrated significantly different abundance patterns (p = 0.017 and p = 0.032, respectively).

**Figure 2:**
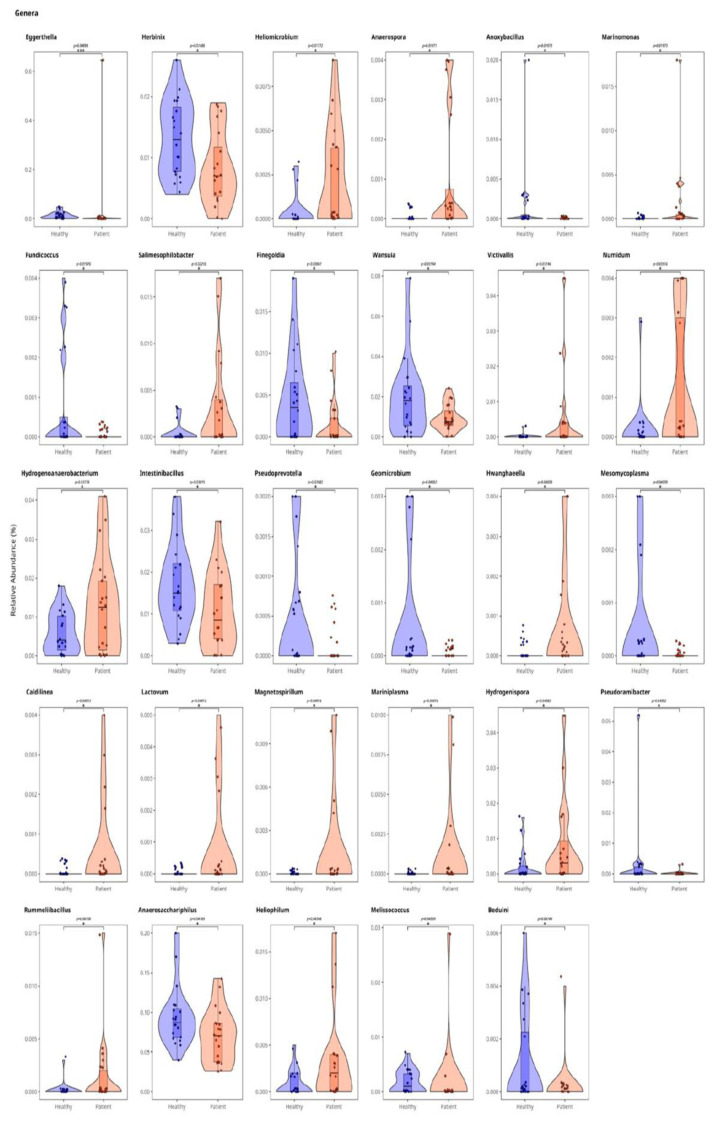
Plots showing the distribution of relative abundances at the genus level across the groups. The violin plots visually represent the distribution, density, and spread of the relative abundance of each species in both groups. Healthy controls are coded in blue, osteoporosis patients are in orange/red. Asterisks (*) and p-values shown above the brackets indicate statistically significant differences between groups. The number of asterisks reflects the level of significance (* p < 0.05, ** p < 0.01, *** p < 0.001), while the exact p-values are additionally reported.

At the genus level, significant differences in gut microbiota composition were observed between the groups (Figure 2). Genera that were significantly increased in the osteoporosis group were *Anaerosporobacter* (p < 0.05), *Hydrogenoanaerobacterium* (p < 0.05), *Magnetospirillum* (p < 0.05), *Hydrogenispora* (p < 0.05), *Veillonella* (p < 0.05), *Finegoldia* (p < 0.05), *Anaerovorax* (p < 0.01), *Ruminiclostridium* (p < 0.05), *Salmonispibacter* (p < 0.05), *Ruminococcus* (p < 0.01), and *Streptococcus* (p < 0.05). Those that were significantly increased in the control group (but were significantly depleted in the osteoporotic group) were *Eggerthella* (p < 0.01), *Herbinix* (p < 0.01), *Intestinibacillus* (p < 0.05), *Geomicrobium* (p < 0.01), *Pseudoprevotella* (p < 0.01), *Lactonifactor* (p < 0.05), *Anoxybacillus* (p < 0.05), *Wasuia* (p < 0.05), *Hydrocarboniphaga* (p < 0.05), and *Beduini* (p < 0.05).

**Figure 3:**
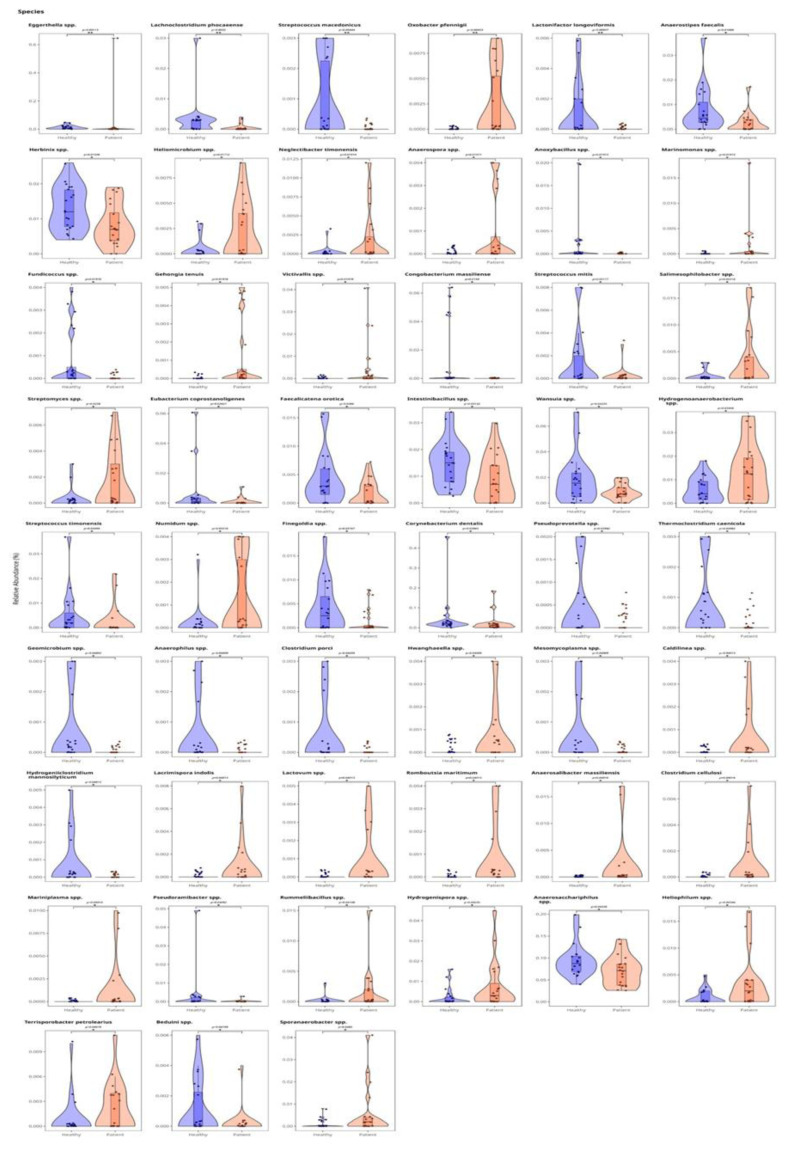
Plots showing the distribution of relative abundances at the genus level across the groups. The violin plots visually represent the distribution, density, and spread of the relative abundance of each species in both groups. Healthy controls are coded in blue, osteoporosis patients are in orange/red. Asterisks (*) and p-values shown above the brackets indicate statistically significant differences between groups. The number of asterisks reflects the level of significance (* p < 0.05, ** p < 0.01, *** p < 0.001), while the exact p-values are additionally reported.

After FDR correction, 51 species, most of which were from the phylum *Bacillota* and class *Clostridia*, were found to differ significantly between the osteoporotic and control groups. SCFA-producing bacteria, including *Faecalibacterium prausnitzii* (p < 0.05), *Anaerostipes faecalis* (p < 0.05), and *Lactonifactor longoviformis* (p < 0.05), were significantly reduced in the osteoporosis group. Species that were significantly increased in the osteoporosis group were *Oxobacter pfennigii* (p < 0.001), *Streptococcus macedonicus* (p < 0.001), *Magnetospirillum gryphiswaldense* (p < 0.05), *Neglecta bacter cinnamomea* (p < 0.05), *Anaerosporobacter* spp. (p < 0.05), *Salmonispibacter* spp. (p < 0.01), *Pseudoprevotella* spp. (p < 0.05), *Ruminiclostridium* spp. (p < 0.01), *Anaerotruncus* spp. (p < 0.05), *Hydrogenispora* spp. (p < 0.05), *Rummeliibacillus* spp. (p < 0.05), *Anaerovorax rubigenes* (p < 0.05), *Hydrocarboniphaga* spp. (p < 0.05), *Clostridium* spp. (p < 0.05), and *Anaerosphaera massiliensis* (p < 0.05). Those that were significantly decreased in the osteoporotic group were *Eggerthella* spp. (p < 0.001), *Lachnoclostridium phocaeense* (p < 0.001), *Faecalibacterium prausnitzii* (p < 0.05), *Anaerostipes faecalis* (p < 0.05), *Lactonifactor longoviformis* (p < 0.01), *Herbinix* spp. (p < 0.001), *Intestinibacillus* spp. (p < 0.05), *Ligilactobacillus phocaeense* (p < 0.05), *Ruminococcus* spp. (p < 0.05), *Pseudoramibacter* spp. (p < 0.05), *Beduini* spp. (p < 0.05), *Lachnospiraceae indolis* (p < 0.05), and *Lactovum* spp. (p < 0.05) (Figure 3).

Spearman correlation analysis revealed positive associations between *Faecalibacterium prausnitzii* abundance and the femoral neck T-score (r = 0.41 and p = 0.018). Negative correlations were noted between *Oxobacter pfennigii* abundance and the lumbar spine BMD (r = −0.43 and p = 0.015).

## 4. Discussion

This study provides novel insights into the relationship between gut microbiota composition and postmenopausal osteoporosis, reinforcing the emerging gut–bone axis paradigm. Using full-length 16S rRNA sequencing with Oxford Nanopore Technology, species-level taxonomic resolution was achieved and significant microbial shifts were identified in the osteoporotic group compared to the control group. These findings align with prior studies suggesting that gut microbiota modulates skeletal integrity through immune, metabolic, and endocrine pathways [16–19]. The osteoporotic group exhibited an enrichment of taxa associated with gut microbiota alterations, gut barrier disruption, and inflammation, and had depleted levels of SCFA-producing and estrogen-modulating species.

The use of full-length 16S rRNA sequencing with Oxford Nanopore Technology represents a major strength of this study, as it enables species-level resolution. Platform-specific limitations are also acknowledged, as nanopore sequencing is known to have a higher raw error rate compared to short-read platforms; however, this limitation was mitigated through stringent quality filtering, length-based read selection, chimera removal, and consensus-based taxonomic assignment. Moreover, recent improvements in nanopore chemistry and basecalling algorithms have substantially enhanced sequencing accuracy, further supporting its suitability for microbiome profiling in clinical research.

Beta diversity analysis revealed significant phylogenetic differences between the groups, which were indicative of evolutionary shifts in microbial communities rather than gross compositional changes. This suggests that subtle, phylogenetically meaningful shifts rather than broad taxonomic changes underpin osteoporosis-associated microbial alterations. Similar findings were reported by Wang et al. [25], indicating restructuring of microbial lineages without overt community disruption. While many taxa were shared between groups, the healthy cohort harbored a higher proportion of unique genera and species—likely reflecting a richer and potentially more protective microbial environment. Conversely, taxa unique to the osteoporotic group may represent opportunistic or proinflammatory species contributing to disturbed metabolic and immune homeostasis.

Consistent with prior studies, a significant depletion of SCFA-producing and estrogen-modulating taxa [26,27,28], notably *F. prausnitzii*, *Anaerostipes faecalis*, and *Lactonifactor longoviformis*, was observed in postmenopausal osteoporotic women. SCFAs, particularly butyrate and propionate, are critical for bone health, inhibiting osteoclastogenesis, supporting osteoblast differentiation, and enhancing calcium absorption by acidifying the gut, increasing mineral solubility, modulating immune responses via G-protein-coupled receptor 41/43 and histone deacetylase inhibition, promoting Treg differentiation and reducing Th17-driven inflammation, facilitating endocrine effects (e.g., increasing IGF-1, leptin, glucagon-like peptide-1), and contributing to bone formation [10,13,16,29]. The positive correlation between *F. prausnitzii* and the femoral neck T-scores supports its clinical relevance. Notably, *L. longoviformis* facilitates the conversion of dietary lignans into enterolignans, potent phytoestrogens like enterolactone with osteoprotective effects [30]. Their depletion may reduce endogenous estrogen-like activity, compounding the effects of menopause-induced estrogen deficiency. Butyrate supplementation improved bone mass and attenuated cytokine levels in ovariectomized mice, reinforcing the importance of these microbial-derived metabolites [31,32]. Some detected taxa may represent transient or low-abundance organisms inherent to nanopore full-length sequencing, but their consistent differential abundance supports biological relevance.

Estrogen deficiency is a known driver of osteoporosis in postmenopausal women, alterations in estrogen-metabolizing microbiota, may contribute to bone loss via impaired enterohepatic circulation and increased systemic inflammation [16]. The increase in *Prevotella* and *Veillonella*, genera previously linked to low-grade inflammation and metabolic dysregulation, could contribute to osteoporotic bone loss through immune-mediated mechanisms [25,29]. These genus-level changes support the notion that osteoporosis is associated with a compositional shift in the gut microbiota that favors a proinflammatory environment detrimental to bone metabolism. Comparative data from Wang et al. [25] link gut metabolomic changes such as glycoursodeoxycholic acid to BMD, while the current study emphasizes species-specific associations. Notably, *O. pfennigii* negatively correlated with lumbar spine BMD, potentially reflecting oxalate metabolism impacting calcium bioavailability.

A key strength of this study is the use of full-length 16S rRNA sequencing, offering high taxonomic precision. However, limitations include the cross-sectional design, small sample size, and absence of functional metagenomics or metabolomics. The complementary use of high-resolution sequencing and metabolomics, as demonstrated by Wang et al. [25], could provide a holistic view of the microbiome–metabolite–bone axis. It is also worth noting that the gut microbiota appears to influence bone metabolism differently depending on the osteoporosis subtype. For example, estrogen deficiency-related osteoporosis is strongly microbiota-dependent, while age-related bone loss may be less influenced by microbial composition [18]. This heterogeneity underscores the need to investigate skeletal site- and phenotype-specific interactions between the gut microbiota and bone health.

This study had several limitations that should be acknowledged. First, the relatively small sample size limited the generalizability of the findings and the statistical power to detect subtle microbial differences. Therefore, the results should be interpreted as exploratory and hypothesis-generating.

Second, due to the cross-sectional study design, causal relationships between gut microbiota alterations and BMD could not be established. Longitudinal and interventional studies are required to clarify the directionality and causality of these associations.

Despite these limitations, the strengths of this study include the use of high-resolution full-length 16S rRNA sequencing and the integration of microbiota data with clinically relevant bone density measurements. Future longitudinal and interventional studies incorporating functional metagenomics, metabolomics, and controlled dietary assessments are warranted to clarify causality and to explore microbiota-targeted strategies for osteoporosis prevention and management.

## 5. Conclusion

This study identified a distinct microbial signature in postmenopausal women with osteoporosis, characterized by reduced SCFA and estrogen-modulating bacteria and enrichment of potentially deleterious, proinflammatory taxa. These alterations may impair mineral absorption, disrupt hormonal metabolism, and promote systemic inflammation, collectively contributing to bone loss. The findings propose that gut microbiota composition could serve as a biomarker for osteoporosis risk and a modifiable therapeutic target. Future longitudinal and interventional studies integrating metabolomics, functional metagenomics, and immune profiling are necessary to establish causality and need to evaluate microbiota-modulating strategies (e.g., probiotics, prebiotics, dietary interventions) which represent promising avenues for osteoporosis prevention and management. is mandatory and should contain the main conclusions regarding the research.

Taken together, these findings highlight the potential of gut microbiota composition as a future biomarker and therapeutic target in postmenopausal osteoporosis; however, further validation in longitudinal and intervention-based studies is required before clinical application.

## Figures and Tables

**Table:** Comparison of clinical, demographic, and BMD parameters between postmenopausal women with osteoporosis and healthy controls.

	Osteoporosis (n=20)	Control (n=20)	p-value
**Age (year)**	60.3±7.10	60.90±8.47	0.810
**Menopause duration (year)**	12.40±8.25	14.05±8.69	0.542
**Age at menopause (year)**	47.90±4.71	46.85±4.94	0.496
**Weight (kg)**	70.65±12.69	74.15±11.5	0.367
**BMI (kg/m** **^2^** **)**	28.50±5.39	30.00±5.20	0.377
**Femur BMD (g/cm** **^2^** **)**	0.78±0.13	0.99±0.09	**<0.001**
**Femur t score**	−1.73±0.95	−0.21±0.67	**<0.001**
**Femur Z score**	−1.01±1.10	0.50±0.97	**<0.001**
**Spine BMD (g/cm** **^2^** **)**	0.70±0.11	1.03±0.10	**<0.001**
**Spine t score**	−3.10±0.82	−0.20±0.88	**<0.001**
**Spine Z score**	–1.81±0.81	1.18±0.84	**<0.001**
**Height shortening (cm)**	3.40±2.39	2.60±2.39	0.398
**Sleep duration**	7.55±1.90	7.60±1.78	0.698

Data are presented as the mean ± standard deviation for continuous variables and as the number (percentage) for categorical variables. Height shortening was calculated as the difference between maximum lifetime height and current height (cm). Bold values indicate statistically significant results.

## References

[1] Sözen T Özışık L Başaran NÇ An overview and management of osteoporosis European Journal of Rheumatology 2017 4 1 46 56 10.5152/eurjrheum.2016.048 28293453 PMC5335887

[2] Smit AE Meijer OC Winter EM The multi-faceted nature of age-associated osteoporosis Bone Reports 2024 20 101750 10.1016/j.bonr.2024.101750 38566930 PMC10985042

[3] Eastell R Szulc P Use of bone turnover markers in postmenopausal osteoporosis Lancet Diabetes Endocrinology 2017 5 11 908 923 10.1016/S2213-8587(17)30184-5 28689768

[4] Kanis JA Cooper C Rizzoli R Reginster JY Scientific Advisory Board of the European Society for Clinical and Economic Aspects of Osteoporosis (ESCEO) and the Committees of Scientific Advisors and National Societies of the International Osteoporosis Foundation (IOF) European guidance for the diagnosis and management of osteoporosis in postmenopausal women Osteoporosis International 2019 30 1 3 44 10.1007/s00198-018-4704-5 30324412 PMC7026233

[5] Li Z Wang Q Huang X Wu Y Shan D Microbiome’s role in musculoskeletal health through the gut-bone axis insights Gut Microbes 2024 16 1 2410478 10.1080/19490976.2024.2410478 39387683 PMC11469435

[6] Lu L Chen X Liu Y Yu X Gut microbiota and bone metabolism FASEB Journal 2021 35 7 e21740 10.1096/fj.202100451R 34143911

[7] Liu J Tan Y Cheng H Zhang D Feng W Functions of Gut Microbiota Metabolites, Current Status and Future Perspectives Aging and Disease 2022 13 4 1106 1126 10.14336/AD.2022.0104 35855347 PMC9286904

[8] Hays KE Pfaffinger JM Ryznar R The interplay between gut microbiota, short-chain fatty acids, and implications for host health and disease Gut Microbes 2024 16 1 2393270 10.1080/19490976.2024.2393270 39284033 PMC11407412

[9] Indrio F Salatto A Gut Microbiota-Bone Axis Annals of Nutrition and Metabolism 2025 81 Suppl 1 47 56 10.1159/000541999 39848230

[10] Zaiss MM Jones RM Schett G Pacifici R The gut-bone axis: how bacterial metabolites bridge the distance The Journal of Clinical Investigation 2019 129 8 3018 3028 10.1172/JCI128521 31305265 PMC6668676

[11] Behera J Ison J Tyagi SC Tyagi N The role of gut microbiota in bone homeostasis Bone 2020 135 115317 10.1016/j.bone.2020.115317 32169602 PMC8457311

[12] Xiong RG Zhou DD Wu SX Huang SY Saimaiti A Health benefits and side effects of short-chain fatty acids Foods 2022 11 18 2863 10.3390/foods11182863 36140990 PMC9498509

[13] Lucas S Omata Y Hofmann J Böttcher M Iljazovic A Short-chain fatty acids regulate systemic bone mass and protect from pathological bone loss Natura Communications 2018 9 1 55 10.1038/s41467-017-02490-4 PMC5754356 29302038

[14] Hrncir T Gut microbiota dysbiosis: triggers, consequences, diagnostic and therapeutic options Microorganisms 2022 10 3 578 10.3390/microorganisms10030578 35336153 PMC8954387

[15] Chen Y Wang X Zhang C Liu Z Li C Gut microbiota and bone diseases: a growing partnership Frontiers in Microbiology 2022 13 877776 10.3389/fmicb.2022.877776 35602023 PMC9121014

[16] Xu Q Li D Chen J Yang J Yan J Crosstalk between the gut microbiota and postmenopausal osteoporosis: Mechanisms and applications International Immunopharmacology 2022 110 108998 10.1016/j.intimp.2022.108998 35785728

[17] Seely KD Kotelko CA Douglas H Bealer B Brooks AE The human gut microbiota: a key mediator of osteoporosis and osteogenesis International Journal of Molecular Sciences 2021 22 17 9452 10.3390/ijms22179452 34502371 PMC8431678

[18] Zheng XQ Wang DB Jiang YR Song CL Gut microbiota and microbial metabolites for osteoporosis Gut Microbes 2025 17 1 2437247 10.1080/19490976.2024.2437247 39690861 PMC11657146

[19] Ma Z Liu Y Shen W Yang J Wang T Osteoporosis in postmenopausal women is associated with disturbances in gut microbiota and migration of peripheral immune cells BMC Musculoskeletal Disorders 2024 25 1 791 10.1186/s12891-024-07904-1 39375626 PMC11460084

[20] Qi P Xie R Liu H Zhang Z Cheng Y Mechanisms of gut homeostasis regulating Th17/Treg cell balance in PMOP Frontiers in Immunology 2024 15 1497311 10.3389/fimmu.2024.1497311 39735544 PMC11671525

[21] Wei J Liu Q Yuen HY Lam AC Jiang Y Gut-bone axis perturbation: Mechanisms and interventions via gut microbiota as a primary driver of osteoporosis Journal of Orthopaedic Translation 2025 50 373 387 10.1016/j.jot.2024.11.003 40171106 PMC11960541

[22] Kanis JA on behalf of the World Health Organization Scientific Group Technical Report World Health Organization Collaborating Centre for Metabolic Bone Diseases, University of Sheffield UK 2007 2007 Assessment of osteoporosis at the primary health-care level.

[23] Oksanen J Blanchet FG Kindt R Legendre P Minchin PR 2014 Vegan: Community Ecology Package R Package Version 4.3-1

[24] McMurdie PJ Holmes S phyloseq: an R package for reproducible interactive analysis and graphics of microbiome census data PLoS One 2013 8 4 e61217 10.1371/journal.pone.0061217 23630581 PMC3632530

[25] Wang Z Wang W Wang Y Hu H Wang B Mapping gut microbiota and metabolite alterations in patients with postmenopausal osteoporosis in the Beijing Community of China European Journal of Medical Research 2025 30 1 539 10.1186/s40001-025-02795-x 40597307 PMC12211186

[26] Li S Wang J Zhang Y Wang J Zhou T Gut microbiota and short-chain fatty acids signatures in postmenopausal osteoporosis patients: A retrospective study Medicine (Baltimore) 2024 103 47 e40554 10.1097/MD.0000000000040554 39809201 PMC11596502

[27] Liu C Yang YN Guo S Cao Y Wang LN Gut microbiota-mediated regulation of skeletal development: A review of mechanistic analysis and interventional strategies Journal of Advanced Research 2025 S2090-1232(25)00557-0 10.1016/j.jare.2025.07.033 PMC13001057 40691987

[28] Kverka M Stepan JJ Associations among estrogens, the gut microbiome and osteoporosis Current Osteoporosis Reports 2024 23 1 2 10.1007/s11914-024-00896-w 39585466 PMC11588883

[29] Chu X Xing H Chao M Xie P Jiang L Gut microbiota modulation in osteoporosis: probiotics, prebiotics, and natural compounds Metabolites 2025 15 5 301 10.3390/metabo15050301 40422878 PMC12113025

[30] Bess EN Bisanz JE Yarza F Bustion A Rich BE Genetic basis for the cooperative bioactivation of plant lignans by *Eggerthella lenta* and other human gut bacteria Nature Microbiology 2020 5 1 56 66 10.1038/s41564-019-0596-1 PMC6941677 31686027

[31] Lyu Z Hu Y Guo Y Liu D Modulation of bone remodeling by the gut microbiota: a new therapy for osteoporosis Bone Research 2023 11 1 31 10.1038/s41413-023-00264-x 37296111 PMC10256815

[32] Zhao Y Wang J Xu L Xu H Yan Y Beyond bone loss: a biology perspective on osteoporosis pathogenesis, multi-omics approaches, and interconnected mechanisms Biomedicines 2025 13 6 1443 10.3390/biomedicines13061443 40564162 PMC12190919

